# UHPLC-DAD Characterization of *Origanum vulgare* L. from Atacama Desert Andean Region and Antioxidant, Antibacterial and Enzyme Inhibition Activities

**DOI:** 10.3390/molecules26072100

**Published:** 2021-04-06

**Authors:** Claudio Parra, Patricio Muñoz, Luis Bustos, Felipe Parra, Mario J. Simirgiotis, Hugo Escobar

**Affiliations:** 1 Laboratorio de Química Orgánica y Productos Naturales, Facultad de Ciencias Agronómicas, Universidad de Tarapacá, Av. General Velásquez 1775, Arica 1000000, Chile; pmunozt@ucdavis.edu (P.M.); luisbustosg@gmail.com (L.B.); filyparramontes@hotmail.com (F.P.); 2 Life Sciences Innovation Center, University of California Davis Chile, Santiago 7511303, Chile; 3 Facultad de Ciencias, Instituto de Farmacia, Universidad Austral de Chile, Valdivia 5110566, Chile; mario.simirgiotis@uach.cl

**Keywords:** α-amylase, α-glucosidase, arid region, bioactive compounds, UHPLC-DAD, oregano

## Abstract

The Lamiaceae family is an important source of species among medicinal plants highly valued for their biological properties and numerous uses in folk medicine. *Origanum* is one of the main genera that belong to this family. The purpose of the study was to determine the phenolic composition of the *Origanum vulgare* extract and evaluate the antimicrobial, antioxidant, and inhibitory activities of this species that grows in the Andean region of the Atacama Desert. High-performance liquid chromatography was performed to determine the main phenols. Rosmarinic acid was identified as the predominant phenolic compound in this species (76.01 mg/100 g DW), followed by protocatechuic acid, which to our knowledge, no previous study reported similar concentrations in *O. vulgare*. The oregano extract exhibited a content of total phenolic (3948 mg GAE/100 g DW) and total flavonoid (593 mg QE/100 g DW) with a higher DPPH antioxidant activity (IC_50_ = 40.58 µg/mL), compared to the same species grown under other conditions. Furthermore, it was found to inhibit α-glucosidase activity with an IC_50_ value (7.11 mg/mL) lower than acarbose (129.32 mg/mL). *Pseudomonas syringae* and *Pantoea agglomerans* (both MIC 0.313 mg/mL and MBC 1.25 mg/mL) were the bacteria most susceptible to oregano extract with the lowest concentration necessary to inhibit bacterial growth. These results open the door for the potential use of this plant to manage chronic diseases, and they expand the knowledge of the species cultivated in arid environmental conditions.

## 1. Introduction

Natural products of plant origin are increasingly used in different industries, including cosmetics, pharmaceuticals, and food. In recent decades, interest has increased in knowing the chemical composition and biological activities of these products since many of the compounds present are involved in protecting human health. For example, phenolic compounds play a vital role in the neutralization and inhibition of free radicals [1]. These compounds also show a broad spectrum of pharmacological properties such as antimicrobial, anti-inflammatory, anticholinesterase, and cardioprotective activities, among others [2,3]. Today, many aromatic and medicinal plants have become attractive to scientists as natural sources of bioactive phytocomplexes and compounds that could be safer than the synthetic compounds used in the industry.

**Figure 1:**
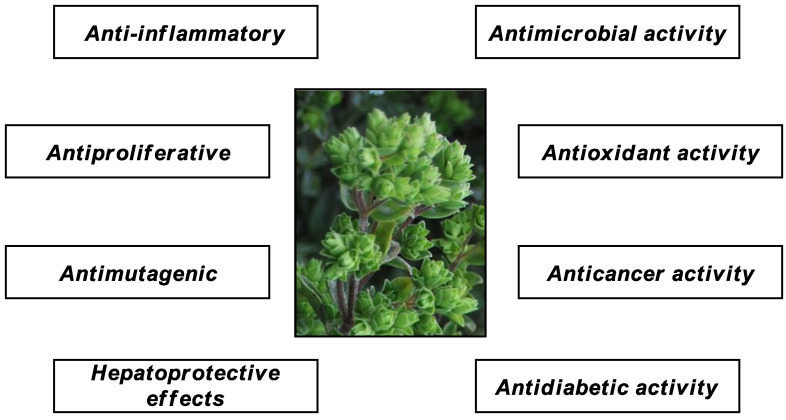
Pharmacological properties of *O. vulgare*.

Plants belonging to the genus *Origanum* (Lamiaceae family) have been known as culinary and medicinal plants. Some species, including *Origanum vulgare* L., are rich in essential oils. *O. vulgare* L. is an aromatic and perennial subshrub and is widely distributed throughout the world. This species is considered extremely variable, both in its morphological characteristics and its chemical composition. The adaptation of *O. vulgare* L. to different edaphological and geographic conditions has given it unique characteristics of biosynthesis of different secondary metabolites depending on the growing environment. Research on new properties of oregano has shown many therapeutic properties: anticancer [4], antimicrobial [5], antioxidant [6], anti-inflammatory [7], antiproliferative [7,8], antimutagenic [9], hepatoprotective effect [10], antidiabetic activity [3], among others (Figure 1). Much of the literature on chemical research on O. vulgare considers its essential oils, and the information on its phenolic compounds is very poor. However, phenolic compounds such as flavonoids and phenolic acids constitute one of the most important pharmacologically active substances that can act as antioxidant, antimicrobial, and anti-inflammatory tools in cells [11,12] and inhibiting low-density lipoproteins’ oxidation [13]. The adaptation of O. vulgare to different edaphological and geographic conditions has given it unique characteristics of biosynthesis of different secondary metabolites depending on the growing environment.

The *Origanum vulgare* L. was introduced more than 100 years ago in Chile, adapting to its unique conditions. One of the oregano growing areas is in the arid region of the Atacama Desert [5]. This growing region is located between 2800 and 3500 m above sea level and presents extreme characteristics for this species’ development (low oxygen availability, high solar radiation, water scarcity, sudden changes in temperature during the day and at night) [14,15]. Traditionally, the leaves and flowers of oregano are used by local communities, mainly for their beneficial properties to cure coughs and sore throats and relieve digestive discomfort [7]. However, *O. vulgare* L. from this area is characterized by a limited distribution and scarce raw material sources. In this context, the geographical and environmental conditions of this region could grant these plants unique properties. Therefore, the present research aims to provide the phenolic composition and antimicrobial, antioxidant, and inhibitory activities of *O. vulgare* L. from the Atacama Desert Andean region (ADAR). These results could open a potential interest in the food and pharmaceutical industry, which are always in search of the new bioactive sources.

## 2. Results and Discussion

### 2.1. Chemical Composition

**Figure 2:**
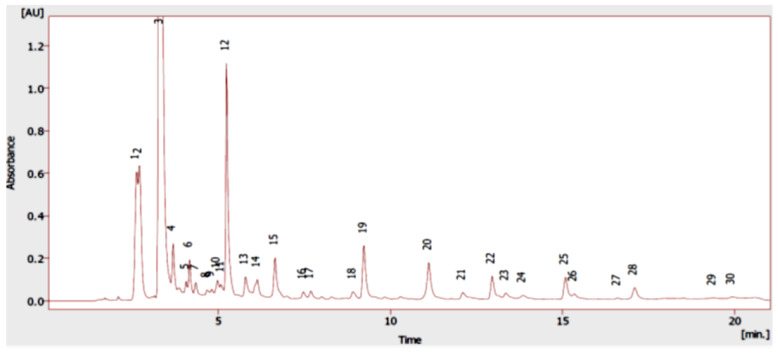
UHPLC-DAD chromatogram of *Origanum vulgare* L. ethanolic extract at 280 nm.

The composition of the *Origanum vulgare* L. extract was analyzed by UHPLC-DAD (Figure 2). By comparing the relative retention times with the commercial standards, the oregano extract compounds could be identified. In total, twenty compounds were identified in oregano extract, and they were mostly phenolic acids (peaks 1, 2, 6, 8, 10, 11, 12, 13, 14, 16, and 18), flavonoids (peaks 4, 5, 15, 19, 20, 23, and 24), and phenolic monoterpenes (peaks 27 and 28).

Table 1 shows the concentrations of the phenolic compounds identified in the analyzed sample. The most abundant phenolic acid was rosmarinic acid (76.01 mg/100 g DW), followed by protocatechuic acids (61.99 mg/100 g DW), gallic (43.60 mg/100 g DW), and ferulic (11.72 mg/100 g DW), while chlorogenic acid was found in lower concentrations. In flavonoids, the most abundant compounds were luteolin and naringenin (27.31 and 24.32 mg/100 g DW, respectively). Carvacrol and thymol were also identified. The concentration of phenolic monoterpenes, especially carvacrol, is similar to that reported for the same species’ essential oil, where low amounts of this compound were found [5]. The presence of these phenolic compounds could be related to the medicinal properties attributed to oregano. In this context, rosmarinic acid (RA) is the major phenolic acid in several Lamiaceae family plants. Several biological activities are attributed to this compound, so RA has focused on many studies [16].

On the other hand, to our knowledge, no previous works have reported similar concentrations of protocatechuic acid in *O. vulgare* L. Protocatechuic acid has a wide range of biological and pharmacological activities, including antioxidant, antibacterial, anticancer, antiulcer, antidiabetic, among others [17,18,19]. The other main phenolic acid in ADAR’s *O. vulgare* L. is gallic acid which has potent antimicrobial, anti-inflammatory and anticancer activities [19]. The main flavonoid was luteolin which is recognized for its anti-inflammatory [20], and antioxidant [21] properties. These characteristics could be associated with the traditional use as anti-inflammatory of this species.

In previous reports on oregano, different concentrations of the compounds found in OV ADAR are reported. These differences can be attributed to this species’ chemical composition that depends on the climate, altitude, harvest time, growth stage, and different extraction methods and experimental protocols. However, these characteristics give the great potential to oregano grown in the Atacama Desert Andean region (ADAR), which could be considered an important natural source of these phenolic compounds. This preliminary characterization of phenolic compounds can help broaden the therapeutic and industrial potential of this species.

### 2.2. Chemical Constituents and Antioxidant Activity of Origanum vulgare L.

Oregano varieties are characterized by different qualitative and quantitative compositions of phenolic compounds. Table 2 shows the total phenolic content (TPC), total flavonoid content (TFC), and antioxidant capacity (DPPH, ABTS, and FRAP) of the *O. vulgare* L. from ADAR. The total phenols content (TPC) for this plant was 3948 ± 40.17 mg GAE/100 g DW, being higher than those reported for *Thymus citriodorus* L. (1797.28 ± 51.45 mg GAE/100 g DW) [22], and also to the informed for Rhubarb (*Rheum* spp.) that is a plant characterized by its very high antioxidant properties [23]. Furthermore, a higher amount of TPC was observed in oregano from the Atacama than for the same species cultivated in other world regions [24,25]. While the total flavonoid content (593 ± 30.50 mg QE/100 g DW), were also close to the values reported for the aerial parts of the oregano from northern Iran (521 to 466 mg QE/100 g DW) [26]. FRAP analysis revealed oregano extract as showing the strongest reducing power than Rheum rhabarbarum [23]. The antioxidant activity values in the ABTS test was 8266 ± 251.50 μmol TE/100 g of DW, being greater than those informed for Macedonia’s medicinal plants, such as Alcea pallida and Saponaria officinalis [27], as well for the Rhubarb [23]. These antioxidant results expand the knowledge of *O. vulgare* L. species cultivated under environmental conditions typical of the Atacama Desert’s Andean region. The free radical scavenging activity of DPPH followed the same trend as the essential oil of *O. vulgare* L. [5]. The DPPH test revealed that the oregano extract (40.58 ± 5.41 µg/mL) showed more DPPH scavenging capacity than the report by Teixeira et al. for the same species (64.1 ± 1.2 µg/mL) [28]. However, the DPPH value found in this work was less than standards Quercetin and Trolox but were close to those reported for some Chilean Nolana species (from 30 to 120 µg/mL) [29].

### 2.3. Antibacterial Activity

**Figure 3:**
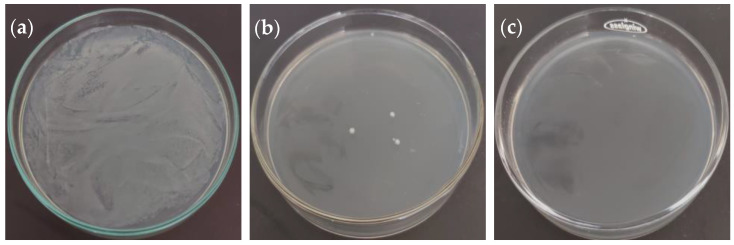

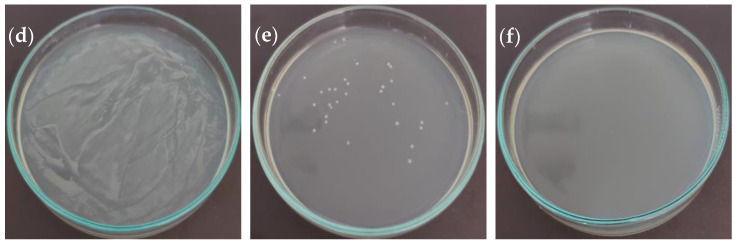
Petri dishes of *Escherichia coli* and *Pseudomonas syringae* at non-inhibitory concentrations (**a** and **d**, respectively), at MIC (**b** and **e**, respectively), and at MBC (**c** and **f**, respectively).

The inhibitory and bactericidal activities of the oregano extract are shown in Table 3. Most bacteria were susceptible to the oregano extract, with a variation in the minimum inhibitory concentration (MIC) values from 0.078 mg/mL to 0.625 mg/mL and minimum bactericidal concentration (MBC) values from 1.25 mg/mL to 5.00 mg/mL (Figure 3). *Xanthomonas campestris* was resistant to the antimicrobial activity of oregano extract. *Agrobacterium tumefaciens* was the most resistant bacterium to the antibacterial activity of oregano extract, showing the highest minimum inhibitory and minimum bactericide concentrations (0.625 mg/mL and 5.00 mg/mL, respectively). *Pseudomonas syringae* and *Pantoea agglomerans* were the most susceptible phytopathogenic bacteria to oregano extract with the lower concentration necessary to inhibit bacterial growth (0.313 mg/mL MIC and 1.25 mg/mL MBC).

On the other hand, *Salmonella enterica* was the most susceptible to oregano extract (0.078 mg/mL MIC and 1.25 mg/mL MBC). MIC and MBC assays using 0–20 µg/mL of kanamycin were carried out as a positive control for each bacterium (Table 3). Bacteria inoculated in nutrient broth with no-extract was used as a negative control to observe microbial growth.

It was not possible to determine MBC for *Bacillus subtilis* due to its ability to produce spores, which would allow surviving the antibacterial activity of *O. vulgare* L. extracts. Spores are resistance structures formed by some bacterial genera that allow them to survive under different extreme conditions and chemical treatments [30]. However, it was not possible to detect antibacterial activity using the paper disk diffusion method (data not showed), probably due to the inaccuracy and lower sensitivity of this methodology compared to MIC and MBC method [31,32].

Previously, Simirgiotis et al. [5] reported that essential oils of *O. vulgare* L. from the arid Andean Region of Chile have antibacterial activity against pathogenic and phytopathogenic bacteria. However, this work showed some differences in MIC and MBC activity compared to the same plant’s essential oil. The differences could be explained by the composition and concentration of active metabolites, particularly thymol and carvacrol, described as antimicrobial compounds and present in lower quantities in the extract of *O. vulgare* L., in comparison to the essential oil of the same plant (Table 1). This difference could explain the higher concentrations of extract needed to inhibit and kill the different tested bacteria. However, the oregano extract showed activity against *A. tumefaciens* and *P. agglomerans*, which is not observed for essential oils of oregano [5]. Rosmarinic acid has been described as an antimicrobial compound [33], which is present in high concentration in the extract of *O. vulgare* L. and could explain the antibacterial activity against *A. tumefaciens* and *P. agglomerans*. Previously, it has been reported the effectiveness of *O. vulgare* L. essential oil to inhibit microbial growth of different food spoiling yeast [34], which indicates the capacity of oregano oil in the food industry. From this point of view, it is possible to propose using ethanolic extract as an additive, inhibiting the microbial growth of bacteria that could affect the quality of foods, such as *B. subtilis*, *S. enterica*, *S. aureus*, among others. Nonetheless, organoleptic properties, safety and toxicity issues of this extract still need to be evaluated beforehand.

### 2.4. Enzyme Inhibitory Activity of Origanum vulgare L. Extract from ADAR

Obesity, dyslipidemia, glycemic index imbalance, glucose intolerance, or hypertension are early signs of the possible development of chronic diseases such as type 2 diabetes. A possible alternative to reduce postprandial hyperglycemia is the inhibition of key associated enzymes to type 2 diabetes, such as α-amylase and α-glucosidase [35]. To control blood glucose levels, inhibition of these enzymes is an important strategy. However, the main drawback of the inhibitors currently used, such as acarbose and voglibose, is their side effects such as diarrhea, abdominal bloating, and flatulence [36]. In the α-amylase and α-glucosidase inhibition, synergy by both terpenoids and phenolics might play crucial roles [37]. The present work determined the IC_50_ values of the extract *O. vulgare* L. on α-amylase, α-glucosidase, and pancreatic lipase (Table 4). OV ADAR’s extract showed a potential effect on the restraint of α-amylase activity (44.71 ± 0.86 µg/mL) and exerted much more powerful α-glucosidase inhibition (7.11 ± 1.37 µg/mL) than Acarbose, a reference compound used in the clinical treatment of diabetes. On the other hand, oregano extract presented a low inhibitory activity on pancreatic lipase (922.35 ± 30.99 µg/mL), compared to positive control Orlistat^®^ [38].

Inhibition of α-amylase was not significant compared to the reference standard, but analysis revealed that oregano extract contains bioactive compounds that can inhibit α-amylase. The activity found for oregano extract on α-glucosidase is higher than that reported for the leaf extract of *Aphloia theiformis*, a medicinal plant used for the management of diabetes (IC_50_ of 55.20 µg/mL for the ethanol extract and 77.63 µg/mL for the leaf decoction, respectively) [39]. However, the pancreatic lipase analysis revealed *O. vulgare* L. as showing the lowest enzymatic inhibition (922.35 µg/mL) that this plant [39]. These results show the potential that OV ADAR could have for the treatment of chronic diseases.

Natural products’ phenolic composition is essential to determine the inhibition of key enzymes associated with metabolic syndrome problems. Several reports indicate that extracts containing rosmarinic acid have inhibitory activity on porcine pancreatic amylase in vitro [40,41]. Besides, herbs containing rosmarinic acid as the main phenolic component have been used in traditional medicine for a long time to treat diabetes *mellitus* or cardiac diseases [42]. Studies on some herbs of the Lamiaceae family have also reported strong α-glucosidase inhibitory activity for some phenolic components, including protocatechuic acid [43]. On the other hand, some works have shown that combinations of gallic acid with acarbose as antidiabetic therapy produce a reduction in acarbose’s side effects [44]. Besides, other compounds detected in our samples, such as luteolin and, carvacrol are also considered antidiabetic molecules [3].

## 3. Materials and Methods

### 3.1. Plant Material

*O. vulgare* L. was collected in the Socoroma town (18°15′4.67″ S; 69°36′7.44″ W, 2945 masl), northern Chile in April 2020 and was identified and deposited in the Herbarium of the Department of Botany of the University of Concepción, Chile (voucher specimen 184934). The oregano sample was dried in a freeze-dryer (Virtis Benchtop model, 3L, Gardiner, NY, USA) for about 48 h with the condenser temperature and chamber vacuum at −50 °C and 12.5 Pa, respectively.

### 3.2. Extraction

Approximately 1 g of the dried OV ADAR was pulverized and then extracted with 300 mL of ethanol 80% *v/v* in the dark in an ultrasonic bath for 30 min, keeping the temperature under control (less than 30 °C) and finally centrifuged. The supernatant was immediately concentrated under vacuum, and a brown extract was obtained (191 mg). For the HPLC analysis, 5 mg of the oregano extract was dissolved in 2 mL of methanol HPLC grade, filtered (PTFE filter), and 10 µL were injected in the instrument. For the TPC, TFC, antioxidant and enzymatic analyses 1 mg/mL of stock solution were used. For the antibacterial assay was used, a 20 mg/mL stock solution was prepared in ethanol.

### 3.3. Chemicals

HPLC solvents and LC formic acid were from Merck (Santiago, Chile). HPLC standards, (−)-epigallocatechin (EPIG), (+)-catechin (CAT), 4-hydroxybenzoic acid (4-HBA), apigenin (API), caffeic acid (CA), carvacrol (CAR), chlorogenic acid (CLA), ferulic acid (FA), gallic acid (GA), kaempferol (KAM), luteolin (LUT), naringenin (NAR), *p*-coumaric acid (PCA), protocatechuic acid (PRCA), rosmarinic acid (RA), rutin (RUT), sinapic acid (SA), trans-cinnamic acid (CIN), thymol (THY), and vanillic acid (VA); all standards with purity higher than 95% by HPLC) were purchased either from Sigma Aldrich (Santiago, Chile). Ethanol absolute, hydrochloric acid, sodium acetate trihydrate, anhydrous sodium carbonate, sodium potassium tartrate, sodium nitrate, sodium hydroxide, aluminum chloride hexahydrate, ferric chloride hexahydrate, and Folin-Ciocalteu reagent were obtained from Merck (Santiago, Chile). Glacial acetic acid, potassium persulfate, ABTS [2,2′-azinobis (3-ethylbenzothiazoline-6-sulphonate)], *p*-nitrophenyl butyrate, Triton X-100, starch, Acarbose, TPTZ (1,3,5-triphenyltetrazolium chloride), and Trolox (6-hydroxy-2,5,7,8-tetramethylchroman-2-carboxylic acid), 3,5-dinitrosalicylic acid, 4-nitrophenyl-α-D-glucopyranoside, pancreatin from porcine pancreas, lipase from porcine pancreas, α-amylase from porcine pancreas, α-glucosidase from *Saccharomyces cerevisiae* were supplied by Sigma-Aldrich (Santiago, Chile).

### 3.4. Preparation of Standard Solutions

Stock standard solutions of all the phenolics (1 mg/mL) were prepared by separately dissolving the accurate amount of each standard into the HPLC grade methanol with sonication aid (for few minutes). Care was taken to ensure that the water bath temperature during sonication does not increase above 30 °C. The stock solutions were stored in the dark at −20 °C. Working standard solutions that contain all the phenolics were prepared, diluting standard stock solutions with the initial mobile phase. The standard solutions were sonicated/vortexed after preparation and before using (injection in the HPLC) in order to ensure maximal solubilization of each compound in the mixture.

### 3.5. High-Performance Liquid Chromatography of Phenolic Compounds

Liquid chromatography parameters were carried out as described by Soto et al. [45], with some modifications. Analyzes were performed on a Knauer Azura analytical UHPLC system (Knauer, Berlin, Germany), consisting of Azura P 6.1 L pump, 3950 autosamplers, and Azura DAD 2.1 L diode array detector with high sensitivity Knauer. A reversed-phase column (Luna^®^, 100 mm × 4.6 mm, 3.0 µm, Phenomenex, Torrance, CA, USA) was used, and the temperature of the column was kept at 25 °C. The mobile phase consists of two solvents: (A) 1% formic acid in the water, and (B) acetonitrile and the following gradient program was run: at 0 min, the A: B ratio was 95:5; at 3 min, 70:30; at 20 min, 30:70; and at 22 min, 95:5. The system was allowed to run for another 10 min to equilibrate the column before each injection. The flow rate of the mobile phase was 0.7 mL/min. The detection wavelengths were 260 (PRCA, 4-HBA, VA, RUT), 270 (GA, EPIG), 280 (CAT, CIN, NAR, CAR, THY), 320 (CA, PCA, FA, SA), 330 (CLA, RA, KAM) and 360 nm (LUT, API) and DAD was recorded from 200 to 500 nm for peak characterization. Calibration standards were prepared by diluting a concentrated mixture solution with an initial mobile phase in the concentration range of 0.2–20 µg/mL for GA, 0.5–30 µg/mL for RUT, 0.25–10 µg/mL for CIN, and 0.5–20 µg/mL for the rest of phenolics. The most likely identification of phenolics was achieved by comparing the retention times and HPLC spectra of each peak in the sample with those of the respective phenolic compound standards.

### 3.6. Determination of Total Phenol and Flavonoid Contents

Total phenol content was determined using the Folin-Ciocalteau method with some modifications [46]. From the 1 mg/mL stock solution, an aliquot of 50 µL was mixed with 1000 µL of Folin-Ciocalteau reagent (50% *v/v*) and 5000 µL of ethanol at 80% *v/v*. After 5 min of reaction, 250 µL of 20% *w/v* Na_2_CO_3_ was added and brought to a final volume of 8000 µL with 80% *v/v* ethanol. It was then allowed to incubate for 30 min at room temperature. Each sample was measured at 760 nm in a Synergy™ HTX multimodal microplate reader (BioTek Instruments, Inc., Winooski, VT, USA) and compared to a calibration curve using gallic acid as the standard. The total flavonoid content was determined by the aluminum chloride colorimetric method [47]. Briefly, 250 µL of the stock solution was diluted with 750 µL of distilled water and then 250 µL of 10% *w/v* NaNO_2_ solution and allowed to stand for 6 min at room temperature. 500 µL of 10% *w/v* AlCl_3_·6H_2_O solution was added, and the mixture was allowed to stand for 6 min. Then, 250 µL of 1 M NaOH solution and 500 µL of distilled water were added to a final volume of 2500 µL. Each sample was measured at 415 nm in a Synergy™ HTX multimodal microplate reader (BioTek Instruments, Inc., Winooski, VT, USA) and compared to a calibration curve using quercetin as the standard.

### 3.7. Antioxidant Activity Assays

Because the antioxidant capacity cannot be described with a single method, *O. vulgare* L. extract’s antioxidant capacities were assayed with three different assays: free radical scavenging (DPPH and ABTS) and reducing power (FRAP) assays. These methods are based on the single electron transfer (SET) reaction, displayed through a change in color as the oxidant is reduced, and hydrogen atom transfer (HAT), which measures the activity of the antioxidant to scavenge peroxyl radicals [28].

#### 3.7.1. Ferric Reducing Antioxidant Power (FRAP)

The ferric reducing antioxidant power of the samples was determined according to described by Parra et al. [46], with some modifications. In a 96-well microplate, 25 µL of stock solution was mixed with 175 µL of FRAP reagent. The mixture was incubated for 15 min at 37 °C, and absorption was recorded at 593 nm in a Synergy™ HTX multimodal microplate reader (BioTek Instruments, Inc., Winooski, VT, USA). Data were expressed as µmol TE/100 g DW. Trolox is used as standard and ethanol 80% *v/v* as blank.

#### 3.7.2. Free Radical Scavenging (DPPH)

The radical DPPH methodology was used with some modifications [47]. An aliquot (150 µL) of different concentrations of extract (6.25–400 µg/mL) was mixed with 50 µL of 1 mM DPPH solution and incubated for 30 min at 25 °C. The absorbance was fixed at 517 nm, shaking for 5 min, and incubation for 30 min at 36 °C. The percentage of radical inhibition DPPH was calculated according to Equation (1):

(1) $$\%\;=\;\frac{\left({Abs}_{control}\;-\;{Abs}_{sample}\right)}{{Abs}_{control}}\;\times \;100$$

Subsequently, a curve of % inhibitory activity of DPPH versus concentration of the extract was drawn, and the IC_50_ value was calculated.

#### 3.7.3. ABTS Method

The ABTS assay was performed by bleaching the cationic radical ABTS^•+^ as described by Echiburu-Chau et al. [47]. The radical ABTS solution was diluted with absolute ethanol until an initial absorbance of approximately 0.70 ± 0.03 was obtained at 734 nm. The radical discoloration was initiated by adding 20 µL of the stock solution to 200 µL of the ABTS^•+^ solution. After 7 min of incubation at 25 °C, the absorbance was measured at 734 nm. Results were expressed as micromoles of Trolox equivalents per 100 g of dry sample (µmol TE/100 g DW).

### 3.8. Antibacterial Activity

#### 3.8.1. Strain and Growth Conditions

Oregano extract was used to determine antibacterial activity against human pathogenic bacteria *Bacillus subtilis* (ATCC 6051), *Escherichia coli* (ATCC 23716), *Salmonella enterica* (ATCC 13311), *Pseudomonas aeruginosa* (ATCC 19429), and *Staphylococcus aureus* (ATCC 29737); and the phytopathogenic bacteria *Agrobacterium tumefaciens* (ATCC 19358), *Erwinia rhapontici* (MK883065), *Pantoea agglomerans* (MK883087), *Pseudomonas syringae* (MF547632), *and Xanthomonas campestris* (MH885473). Bacteria were inoculated into nutrient broth containing 5.0 g/L peptone and 3.0 g/L meat extract and incubated at 25 °C (plant pathogens) or 35 °C (human pathogens) for 18 h at 150 rpm using an incubator with orbital shaking LOM-80 (MRC Lab, London, UK).

#### 3.8.2. Minimum Inhibitory Concentration (MIC)

The MIC of oregano extract was determined by the method described by Simirgiotis et al. [5] in order to determine the minimum concentration necessary to inhibit bacteria. Working concentrations ranged from 0 to 10 mg/mL of oregano extract. For this purpose, a stock solution of 20 mg/mL of extract. Further dilutions were prepared in nutrient broth previously to inoculate with each bacterium. Each dilution was prepared to a final working volume of 200 µL in 96-well plates and inoculated with the different bacteria to be tested at 25 °C or 35 °C, as appropriate. Nutrient broth without extract was inoculated with each bacterium used as growth control; nutrient broth with 0–10 mg/mL of oregano extract without bacteria was employed as a negative control of growth sterility of the oregano dilution. An assay using 0–90% (*v/v*) ethanol was performed to estimate the ethanol MIC for each bacterium. Concentrations higher than 20% ethanol was inhibitory for all bacteria. A 40 mg/mL oregano extract was prepared as a stock solution to avoid ethanol’s inhibitory activity during these assays. After 24 h of incubation, the MIC was determined from the lowest extract concentration where no bacterial growth was observed.

#### 3.8.3. Minimum Bactericidal Concentration (MBC)

The MBC of oregano extract was determined from the last three wells where no bacterial growth was observed in the MIC assay, as described by Simirgiotis et al. [5]. For this, 100 µL from these wells were taken and plated in nutrient broth plates supplemented with 1.5% agar. As a growth control, a culture with no bacterial inhibitory growth in the MIC test was used. The plates were incubated for 24 h at the corresponding temperature, after which the MBC of oregano extract was determined from nutrient agar plates where no growth was observed.

### 3.9. Inhibitory Effect on Metabolic Syndrome-Associated Enzymes

#### 3.9.1. α-Amylase Inhibition Assay

The α-amylase inhibition assay was carried out as described by Burgos-Edwards et al. [48] with slight modifications. Briefly, 100 μL of different concentrations of extract (6.25–200 µg/mL) was co-incubated with 100 µL of 1% *w/v* starch for 5 min at 37°C, and then 100 µL of α-amylase solution (10 U/mL) were added and incubated for a further 30 min. At the end of the incubation, 200 µL of the color reagent were added and incubated for 15 min in boiling water. Then, 40 µL of this mixture was diluted with 210 µL of water, and absorbance was measured in a microplate reader at 565 nm (BioTek Instruments, Inc., Winooski, VT, USA). Acarbose was used as the reference compound.

#### 3.9.2. α-Glucosidase Inhibition Assay

The α-glucosidase inhibition assay was carried out as described by Burgos-Edwards et al. [48] with slight modifications. Briefly, the reaction mixture contained 20 µL of sodium phosphate buffer (200 mM), 120 μL of of different concentrations of extract (6.25–200 µg/mL) prepared in the same buffer and 20 µL of α-glucosidase (0.30 U/L) solution. After 15 min pre-incubation at 37 °C, the reaction was started by adding 20 µL of *p*-nitrophenyl-α-D-glucopyranoside (1 mM) into the wells. The reaction was further incubated for 30 min at 37 °C. Then, absorbance was measured at 415 nm in a microplate reader (BioTek Instrument, Inc., Winooski, VT, USA). Acarbose was used as the reference compound.

#### 3.9.3. Lipase Inhibition Assay

This assay was carried out according to Picot and Mahomoodally [39], with slight modifications. Porcine pancreatic lipase type II was re-suspended in ultrapure water at 20 mg/mL. The substrate *p*-nitrophenyl butyrate (25 mM) was prepared in 5 mM sodium acetate buffer containing 1% Triton X-100. This solution was heated in boiling water for 2 min for a better dissolution and cooled down to room temperature. The assay mixture was 160 µL of 100 mM Tris buffer, 20 µL of sample (31.25–1000 µg/mL), 60 µL of lipase and 180 µL of substrate solution. The mixture was incubated for 60 min at 37 °C and absorbance was read at 405 nm. Orlistat was used as the reference compound.

### 3.10. Statistical Analysis

The statistical analysis was carried out using the originPro 9.1 software packages (Originlab Corporation, Northampton, MA, USA). The determination was repeated at least three times for each sample solution. The Tukey comparison test determined significant differences between means (*p* values < 0.05 were regarded as significant).

## 4. Conclusions

Our study showed that the results obtained for *O. vulgare* L. adapted to the climatic conditions of the Andes of northern Chile contain a series of bioactive substances that are easily extracted using non-toxic, inexpensive, and widely available solvents and that exhibit in vitro activity. This species, grown in an arid area, can produce different secondary metabolites while preserving the typical characteristics of the Mediterranean plant. The chemical composition revealed that this herb is a rich source of rosmarinic acid, a compound known for its extremely high antioxidant properties. Besides, it was demonstrated that protocatechuic acid content was higher than that reported for this same plant cultivated under other conditions. Regarding the results obtained, Atacama oregano contains relatively high levels of phenols and total flavonoids that are essential in the human diet and high concentrations of bioactive substances with antioxidant and antimicrobial properties. Furthermore, the activity found for oregano extract on α-glucosidase opens the door for the potential use of this plant to manage chronic diseases such as diabetes. The cultivation of this herb in extremely arid conditions provides this common food plant with the ability to be a rich source of bioactive substances that can boost its consumption, not only as a spice but also as natural medicine.

## Figures and Tables

**Table 1:** Phenolic compounds identified in the ethanolic extract of *O. vulgare* L.

Peak	Phenolic Compounds	RT	OV ADAR *
**1**	Gallic acid	2.62	43.60 ± 2.91
**2**	Protocatechuic acid	2.70	61.99 ± 3.55
**4**	(−)-epigallocatechin	3.68	4.09 ± 0.04
**5**	(+)-catechin	4.07	2.92 ± 2.14
**6**	4-hydroxybenzoic acid	4.17	9.99 ± 1.07
**8**	Chlorogenic acid	4.67	2.55 ± 0.67
**10**	Vanillic acid	4.97	5.45 ± 0.66
**11**	Caffeic acid	5.08	4.85 ± 0.26
**12**	Rosmarinic acid	5.23	76.01 ± 2.01
**13**	*p*-Coumaric acid	5.78	9.32 ± 0.06
**14**	Ferulic acid	6.13	11.72 ± 0.19
**15**	Rutin	6.65	18.01 ± 2.41
**16**	Sinapic acid	7.47	3.29 ± 0.36
**18**	*trans*-cinnamic acid	8.92	3.82 ± 0.81
**19**	Luteolin	9.23	27.31 ± 2.13
**20**	Naringenin	11.12	24.32 ± 1.15
**23**	Apigenin	13.35	5.81 ± 0.57
**24**	Kaempferol	13.85	5.76 ± 1.24
**27**	Carvacrol	16.60	1.16 ± 1.01
**28**	Thymol	17.08	8.10 ± 2.52
Phenolic acids and their derivatives			232.59 ± 12.55
Flavonoids			88.22 ± 9.68
Phenolic monoterpenes			9.26 ± 3.53

* (mg/100 g of DW); Values are means of three determinations. rt: Retention Time.

**Table 2:** Bioactive compound and antioxidant activities of *Origanum vulgare* from ADAR.

Species	DPPH	ABTS	FRAP	TPC	TFC
	(µg/mL)	(µmol TE/100 g DW)		(mg GAE/100 g DW)	(mg QE/100 g DW)
OV ADAR	40.58 ± 5.41 ^a^	8266 ± 251.50	5156 ± 163.26	3948 ± 40.17	593 ± 30.50
Quercetin *	6.99 ± 0.02 ^b^	-	-	-	-
Trolox *	20.99 ± 1.24 ^c^	-	-	-	-

* Used as standard antioxidants; Values having different superscripts differ significantly (*p* < 0.05).

**Table 3:** Antibacterial activity of *Origanum vulgare* L. extract from ADAR.

Bacteria	MIC ^1^ (mg/mL)	MIC Kan ^2^ (µg/mL)	MBC ^3^ (mg/mL)	MBC Kan ^4^ (µg/mL)
*Escherichia coli* (ATCC 23716)	0.156	5.00	2.50	10.00
*Pseudomonas aeruginosa* (ATCC 19429)	0.156	5.00	1.25	10.00
*Salmonella enterica* (ATCC 13311)	0.078	2.50	1.25	5.00
*Bacillus subtilis* (ATCC 6051)	0.156	1.25	ND	20.00
*Staphylococcus aureus* (ATCC 29737)	0.156	2.50	1.25	10.00
*Erwinia rhapontici* (MK883065)	0.313	1.25	2.50	2.50
*Pseudomonas syringae* (MF547632)	0.313	1.25	1.25	2.50
*Pantoea agglomerans* (MK883087)	0.313	2.50	1.25	5.00
*Agrobacterium tumefaciens* (ATCC 19358)	0.625	1.25	5.00	5.00
*Xanthomonas campestris* (MH885473)	ND	2.50	ND	10.00

^1^ Minimum Inhibitory Concentration. ^2^ Minimum Inhibitory Concentration of kanamycin (positive control). ^3^ Minimum Bactericidal Concentration. ^4^ Minimum Bactericidal Concentration of kanamycin. ND: inhibition not detected. ATCC: American Type Culture Collection (USA). MK883065, MF547632, MK883087, and MH885473 are the accession number to Genbank of the respective bacterium.

**Table 4:** Enzyme inhibitory activity of *O. vulgare* L. extract.

Sample	α-Amylase (IC_50_ µg/mL)	α-Glucosidase (IC_50_ µg/mL)	Pancreatic Lipase (IC_50_ µg/mL)
OV ADAR	44.71 ± 0.86 ^a^	7.11 ± 1.37 ^a^	922.35 ± 30.99 ^a^
Acarbose *	29.49 ± 0.94 ^b^	129.32 ± 1.52 ^b^	-
Orlistat^®^ *	-	-	40.83 ± 3.04 ^b^

* Used as standard drug; Values having different superscripts differ significantly (*p* < 0.05).

## Data Availability

Data is contained within the article.

## Sample Availability

Samples of the compounds are not available from the authors.
